# Effects of Exercise on the Skin Epithelial Barrier of Young Elite Athletes-Swimming Comparatively to Non-Water Sports Training Session

**DOI:** 10.3390/ijerph18020653

**Published:** 2021-01-14

**Authors:** Inês Paciência, Ana Rodolfo, Leonor Leão, Diana Silva, João Cavaleiro Rufo, Francisca Mendes, Patrícia Padrão, Pedro Moreira, Jose Laerte Boechat, Luís Delgado, André Moreira

**Affiliations:** 1 Serviço de Imunologia Básica e Clínica, Departamento de Patologia, Faculdade de Medicina da Universidade do Porto, Al. Prof. Hernâni Monteiro, 4200-319 Porto, Portugal; anaiprodolfo@gmail.com (A.R.); disolha@gmail.com (D.S.); jcrufo@gmail.com (J.C.R.); francisca_castromendes@hotmail.com (F.M.); jl_boechat@id.uff.br (J.L.B.); ldelgado@med.up.pt (L.D.); andremoreira.fmup@gmail.com (A.M.); 2 EPIUnit, Instituto de Saúde Pública da Universidade do Porto, Rua das Taipas 135, 4050-600 Porto, Portugal; padraopatricia@gmail.com (P.P.); spcnaspcna@gmail.com (P.M.); 3 Serviço de Imunoalergologia, Hospital São João, Al. Prof. Hernâni Monteiro, 4200-319 Porto, Portugal; leonorcarneiroleao@gmail.com; 4 Faculdade de Ciências da Nutrição e Alimentação da, Universidade do Porto, 4150-177 Porto, Portugal

**Keywords:** disinfection-by-products, indoor exposure, skin, swimming pool, TEWL, training

## Abstract

The benefits of swimming have been extensively assessed. However, swimming pools contain chlorine and other irritating chemicals that may induce contact dermatitis. To evaluate the effect of a swimming training session on transepidermal water loss (TWEL) in swimmers compared to football players, elite swimmers and football players were invited to participate (58 athletes) in the study, where TEWL was measured before, immediately after, and 30 min after a 2 h training session. The probe was held on the dorsum of the hand, volar forearm, and on the antecubital flexure for 1 min. The volar forearm, antecubital flexure, and hand dorsum showed a significant increase in TEWL in swimmers in both measurements after training compared to baseline (*p* < 0.001). In football players, an increase in TEWL was observed on the hands’ dorsum between baseline and after training measurements. The variations on TEWL levels before and immediately after the training session were higher among swimmers on the volar forearm (*p* = 0.002) and antecubital flexure (*p* = 0.019). Our findings support the effect of the training environment—swimming pool versus outdoor sports—on the skin barrier function, with an increase of transepidermal water loss immediately after exercise. Exposure to a swimming pool environment in a 2 h training session may lead to changes in skin barrier function.

## 1. Introduction

Swimming is frequently recommended by physicians due to the multiple associated benefits, and these recommendations are often made to atopic patients [1]. A review of eight randomized studies evaluated the effectiveness and safety of swimming training as an intervention for asthma in children and adolescents aged 18 years and under, concluded that swimming training was well-tolerated, increasing lung function and cardio-pulmonary fitness [2]. However, swimming has been associated with deleterious effects on competitive swimmer’s health. The high and long-term exposure to a swimming pool environment has been implicated as a potential cause of bronchial barrier dysfunction, mainly due to the high levels of chlorine and chlorination by-products, such as trichloramine, which may induce bronchoconstriction [3,4,5,6].

Additionally, exposure to disinfection by-products (DBP) in swimming pools has been associated with multiple skin disorders, most notably “Swimmer’s xerosis” caused by damage of the stratum corneum [7]. Skin disorders, including eczema, and contact dermatitis, are in fact more frequent in swimmers and workers exposed to swimming pool water [8,9,10]. The duration of lifetime swimming pool attendance has been shown to increase the risk of eczema in children (odds ratio (OR) 1.71, 95% confidence interval (CI) 1.38; 2.12 for >5 years) [10]. An increase in cutaneous symptoms was also reported among pool attendants compared to swimming pool employees [11]. The prevalence of verrucas, mycosis, eczema, and rashes were higher in lifeguards and trainers than other workers at swimming pools [11]. Pardo et al. [12] also reported a dose-response association between the exposure duration to chlorinated swimming pools and the incidence of contact dermatitis among hydro-therapists. Recent studies showed that an increase in transepidermal water loss (TEWL) levels might precede the clinical manifestation of atopic dermatitis in children with a high risk of atopy [13]. Specifically, TEWL may be an important indicator of the integrity of the stratum corneum, with high levels of TEWL being associated with increases in skin irritability [14]. Additionally, a previous study found higher TEWL levels in children with filaggrin-null mutations without atopic dermatitis, suggesting that the TEWL may predict the development of skin diseases [14,15]. The prolonged exposure to a swimming pool environment may contribute to an increase of the skin epithelial barrier permeability and thereby facilitate the penetration of allergens and the development of eczema [16,17]. Therefore, our study aimed to assess the effect on TEWL of a training session on swimmers compared to athletes of non-water sports.

## 2. Materials and Methods

### 2.1. Study Design

This is a non-randomized controlled study. Elite swimmers enrolled in the SWAN trial (Clinical Trials.gov Identifier: NCT03017976) [18] and competitive football players from an international football team were invited to participate. Swimmers training an average of 16 h per week and engaged in competitive training for a minimum period of three years [18]. Football players, considered as a control group, engaged in a training program five sessions a week (each session lasting 2 h) and did not take part in any activities in swimming pools during the study period. In total, 33 regular and competitive swimmers (12–21 years old) from one indoor swimming pool, and 34 football players from a B team, sub-14/15 and sub-13 levels (11–18 years old) were invited to participate. Considering a confidence level of 95%, the estimated statistical power for baseline assessments was higher than 75.0% (94.4% for hand’s dorsum, 75.0% for volar forearm and 82.0% for antecubitis measures). The study was conducted in accordance with the Declaration of Helsinki and after approval by the Ethics Committee of the University Hospital Center of São João, Porto, Portugal. The participants received written information about the purpose of the study and all participants, or their caregivers, have given their written informed consent.

### 2.2. Assessments

A self-administered ISAAC (International Study of Asthma and Allergies in Childhood)-based questionnaire [19] was filled out by participants or by participants’ caregivers comprising sociodemographic and health-related information. Atopic dermatitis was defined as a positive answer to the questions “In the past 12 months, have you ever had itchy skin changes?” and “These skin changes have ever affected any of these parts: the folds of the elbows, behind the knees, front of the ankles, between the buttocks or around the neck, ears or eyes”. Asthma and rhinitis were defined by a self-reported medical diagnosis.

A physical and clinical assessment was also performed by trained health professionals. Height was measured to the nearest 1 mm with a standard stadiometer. Weight was measured to the nearest 0.1 kg, using a digital scale (Tanita™ BC-418 Segmental Body Analyzer, Tanita Corp. Tokyo, Japan). Body mass index (BMI) was calculated using the ratio of weight/height^2^ (kg/m^2^). Skin prick tests were carried out in accordance with international guidelines [20] with a standard battery of commercial extracts for common aeroallergens (house dust mite, mix of weeds, mix of grasses, cat dander, dog dander, and *Alternaria alternata*). Histamine dihydrochloride (10 mg/mL) and diluent were used as positive and negative controls, respectively. Results were recorded after 15 min afterward, and allergic sensitization was defined by a positive skin prick test (SPT) to at least one of the allergens. Lung function and airway reversibility were assessed by spirometry according to the American Thoracic Society criteria [21] using a MasterScreen Vyntus IOS (MasterScreen IOS, Carefusion^®^, Erich Jaeger, Hoechberg, Germany) before and 15 min after 400 μg of salbutamol in aerochamber^®^. TEWL measurements (g/m^2^/h) were performed using a Tewameter^®^TM300 (Courage and Khazaka, Cologne, Germany). All measurements were performed under controlled environmental conditions (room temperature: 20 °C to 23 °C; and relative humidity: 45% to 60%) and after at least 15 min rest period at each time of assessment to avoid interference by environmental temperature or sweating [22]. A shielding box was used to guaranty the absence of undesirable air turbulence; direct and close light sources were also avoided. TEWL was measured on the hand’s dorsum, volar forearm, and the antecubitis by applying the probe to the exposed skin for 1 minute. The TEWL levels were measured before training (T0), immediately after training (T1) and 30 min after training (T2). All participants showered before the last measurements (T2). Participants were advised not to apply emollients before readings and also to gently dry off with a towel before T1 and T2.

### 2.3. Statistical Analysis

The distribution of quantitative variables was determined using the Kolmogorov-Smirnov test. Continuous data were described by the median and interquartile range (IQR). Categorical data are expressed as absolute or percentage frequency. The comparisons between groups were performed using non-parametric tests. The differences between the time of assessment (T0, T1, and T2) in each location were evaluated using a Wilcoxon test. The Mann–Whitney U test was used to evaluate the differences between the time of assessment in both athletes group. The comparison of the variation in the TEWL levels according to the time of assessment (T0, T1, and T2) and body location (hand’s dorsum, volar forearm, and the antecubitis), between swimmers and football players, was performed with One-Way ANOVA, after data logarithmization. Significant differences were reported with an α-value of less than 5% (*p* < 0.05). Statistical analysis was performed using SPSS^®^ statistical package software v21.0 (IBM, New York, NY, USA).

### 2.4. Participants

From the 67 invited athletes, a total of 58 athletes with data on TEWL, including 33 regular and competitive swimmers and 25 professional football players, aged 12 to 21 years, were considered. The study participants’ characteristics are presented in Table 1.

## 3. Results

**Figure 1:**
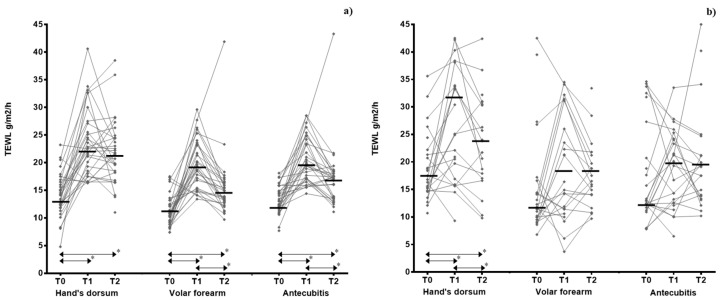
Variation of transepidermal water loss measurements by swimmers (**a**) and football players (**b**), body location, and stages of evaluation. T0—before training, T1—immediately after training, T2—30 min after training with an acclimatization period of 15 min allowed before each measurement. * significant differences (*p* < 0.05).

There was a significant increase in TEWL levels immediately and 30 min after the training in all skin areas among swimmers compared to baseline. The median TEWL levels on hand’s dorsum in football players were also significantly higher immediately and 30 min after the training compared to baseline (T0). Similar trends were also observed for volar forearm and the antecubitis among football players, but no significant differences were found (Table 2, Figure 1).

Comparing both athlete groups, the variations on TEWL levels before and immediately after the training session were significantly higher among swimmers on volar forearm (median (IQR) = 9.25 (5.35; 12.1) versus 4.60 (−2.85; 19.1) g/m^2^/h, *p* = 0.002)) and antecubital flexure (median (IQR) = 6.28 (2.40; 10.4) versus 0.10 (−5.70; 10.1) g/m^2^/h, *p* = 0.019). No differences were observed when considering the variations on TEWL levels before and 30 min after training (Table 2).

## 4. Discussion

In this study, we assessed the effect of a 2 h swimming training session on transepidermal water loss. Our results showed that swimming might be associated with a greater variation in the levels of transepidermal water loss levels, suggesting that exposure to a swimming pool environment may damage the epithelial barrier function, namely the stratum corneum, among elite swimmers.

Our study has a few limitations. The study only included elite swimmers and football players, not allowing the generalization of the results to recreational or non-competitive athletes. There was no control group including recreational swimmers to assess the effect of degree of exposure to chlorinated pool water on TEWL. However, the control group who did not go swimming allowed to evaluate whether the changes in TEWL reflect the exposure to swimming pool environment or exercise-induced changes in skin physiology. Although physical activity has been found to influence skin barrier function [23], we do not expect the differences observed between the two groups of athletes to be due to the type of exercise among athletes within the same sports classification groups based on peak static and dynamic components achieved during competition [24]. This study has only considered the levels of TEWL, and no data on skin pH has been considered. However, changes in TWEL have been previously reported as an important parameter to evaluate the condition and the function of the skin barrier [22].

Our study also presents important strengths. This is the first study assessing the effects of a swimming training session on TEWL, comparing swimmers and athletes of non-water sports. The assessment of the TEWL was performed with an extensively used device, allowing the detection of even subtle skin barrier changes. The levels of TEWL has been previously correlated with stratum corneum damage resulting from exposure to chemicals and also with subjective assessment of irritancy [25]. Additionally, all measures were performed under controlled environmental conditions, reducing the influence of exogenous-related factors on TEWL [22]. We tested different body locations at different moments, and the results support recent findings that skin barrier function develops differently depending on body location [22]. Previous studies reported that TEWL levels vary largely between anatomic sites, which may be related to the corneocyte size [26,27]. As stated by Mayrovitz et al. [28], there is “no optimal TEWL for the entire skin”, but skin areas with larger corneocytes are usually associated with lower TEWL [26,27,29]. However, body location with higher TEWL, such as facial skin, may be more sensitive than other locations (as forearm) to the effects of psychologic stress [30]. The inter-individual and inter-location variability on TEWL values observed among football players may be related to the differences in the equipment used during training. According to Rougier et al. [31], those location sites which are typically covered by clothing may have larger corneocytes compared with sites that are not and consequently associated with the variability of TEWL levels observed among football players. Our study did not consider other anatomic sites, including forehead, legs, abdomen, that may be differently exposed to environment and consequently showing different levels of TEWL. Further studies should be performed to evaluate the long-term effect of exposure to swimming pool on skin barrier function. In addition to the effect of chlorine by-products, future studies should assess the effect of indoor pools sanitized with different chemical disinfectants. The quality of indoor air and pool water was also measured during the same period and was in accordance with the Portuguese regulations for occupational exposure [32]. Additionally, the influence of aging on skin barrier function is widely accepted, and previous studies also reported changes in TEWL levels with age [26,33]. Our results showed a negative correlation (*p* = 0.045) between age and baseline TEWL levels on volar forearm only among swimmers, suggesting that the effects of prolonged and daily exposure to swimming pool environment may increase with age.

A previous study reported no significant differences in TEWL levels after recreational swimming in healthy skin [34]. Authors also reported that levels returned to baseline levels within 24 h after swimming training, suggesting that healthy skin may be able to be restored to homeostasis after exposure to pool water [34]. Garcia Bartels et al. [35] observed a decrease in TEWL levels between the first swimming session (baseline) and at follow-up (eight weeks after the baseline visit and one week after the last swimming session) in infants. Our results suggested that a single swimming pool session leads to a change in TEWL levels among elite swimmers, possibly reflecting changes in skin barrier function and an increased risk of skin diseases/symptoms.

Exposure to DBP has been associated with symptoms of skin dryness and irritation [36], as such compounds may act as membrane-permeant oxidants, disrupting, and increasing the permeability of the epithelial barrier [8,9]. In fact, Gardinier, Guehenneux, Latreille, Guinot, and Tschachler [34] demonstrated that a single swimming pool session leads to a substantial removal of the sebum from the skin surface. Additionally, exposure to a swimming pool environment may promote an increase in the skin’s pH, which can change the skin’s permeability [37], resulting in dry, scaly, and itchy skin [9].

## 5. Conclusions

In conclusion, our study provides support for the potential role of a swimming pool environment and attendance on transepidermal water loss among elite swimmers. Moreover, this study highlights the growing recognition that exposure to DBP may damage the skin barrier.

## Figures and Tables

**Table 1:** Characteristics of participants.

Characteristics	Swimmers	Football Players	*p*-Value
n (males)	33 (10)	25 (19)	0.001 ^1^
Age (years), median (IQR)	14 (13–16)	15 (14–16)	0.383
BMI, kg/m^2^, median (IQR)	20.8 (19.5–22.2)	20.8 (19.4–22.5)	0.588
Asthma, *n* (%)	1 (3.0)	3 (12.0)	0.609
Rhinitis, *n* (%)	10 (30.3)	2 (8.0)	0.018 ^1^
Atopic dermatitis, *n* (%)	2 (6.1)	1 (4.0)	1.000
Allergic sensitization, *n* (%)	16 (48.5)	9 (36.0)	0.182

IQR—interquartile range; BMI—body mass index. ^1^ significant differences (*p* < 0.05).

**Table 2:** Transepidermal water loss values (g/m^2^/h) before, immediately, and 30 min after training.

	Swimmers (*n* = 33)					Football Players (n = 25)					Swimmers vs. Football Players	
	Before (T0)	After (T1)	30′ After (T2)	*p*-Value After	*p*-Value 30′ After	Before (T0)	After (T1)	30′ After (T2)	*p*-Value After	*p*-Value 30′ After	*p*-Value After	*p*-Value 30′ After
Hand’s dorsum	13.7 (11.8–16.3)	22.7 (18.4–27.2)	21.5 (18.2–24.5)	<0.001	<0.001	17.1 (14.7–21.9)	31.8 (17.6–39.8)	24.0 (17.5–31.6)	0.020	0.043	0.279	0.236
Volar forearm	10.7 (9.2–12.9)	19.3 (15.4–23.5)	14.4 (12.9–16.4)	<0.001	<0.001	12.7 (9.9–17.0)	18.8 (11.4–32.0)	18.8 (14.0–25.9)	0.086	0.085	0.002	0.199
Antecubitis	13.1 (11.8–15.6)	19.6 (17.1–24.4)	16.9 (13.6–18.2)	<0.001	<0.001	13.2 (12.2–30.6)	19.7 (12.5–24.9)	19.3 (13.9–24.9)	0.584	0.341	0.019	0.252

Data expressed as median (interquartile ratio(IQR)).

## Data Availability

Data available on request due to restrictions eg privacy or ethical.
